# Gender-Based Differences in Stroke Types and Risk Factors Among Young Adults: A Comparative Retrospective Analysis

**DOI:** 10.3390/jcm14030663

**Published:** 2025-01-21

**Authors:** Sumaira Gulzar, Bushra Hafeez Kiani, Raja Waseem Akram, Ahmed M. Hussein, Abdulaziz Alamri

**Affiliations:** 1 Department of Biological Sciences, Faculty of Sciences, International Islamic University, Islamabad 44000, Pakistan; 2 Department of Public Health Sciences, Health Services Academy, Park Rd, Chak Shahzad, Islamabad 44000, Pakistan; 3 Department of Pharmaceutical Sciences, Division of Pharmacology and Toxicology, University of Vienna, 1090 Vienna, Austria; 4 Biochemistry Department, College of Science, King Saud University, Riyadh 11433, Saudi Arabia

**Keywords:** stroke, gender difference, risk factors, cerebrovascular accident, demographics

## Abstract

**Background/Objectives**: Stroke is considered the second-leading cause of mortality and a primary contributor to adult disability among both men and women. The primary aim of this research is to conduct a comprehensive investigation into gender disparities and stroke subtypes concerning symptoms, risk factors, and clinical and laboratory aspects of stroke, with a specific focus on young stroke patients. **Methods:** In this retrospective comparative study, a total of 185 stroke patients were selected through random sampling from the neurology department of a local hospital in Pakistan between August 2022 and March 2024. Data collection was carried out using a standardized questionnaire, and the collected data were cleaned, processed, input, and analyzed using SPSS software version 24.0. Statistical analysis was performed using Pearson’s chi-square test for categorical variables, and descriptive statistics were utilized to present the frequency, percentages, means, and standard deviations of the variables. Statistical significance was set at a *p*-value of <0.05. **Results:** Out of the 185 participants in this study, 122 (65.9%) were male and 63 (34.1%) were female. The comparison of laboratory, clinical, and risk factors between males and females revealed a higher prevalence of smoking in males compared to females (*p* = 0.014). Additionally, higher levels of LDL and triglycerides were noted in males, while females showed a greater prevalence of vertigo (*p* = 0.002). No statistically significant differences were found in the comparison of laboratory and clinical characteristics among stroke types. In ischemic stroke patients, significant associations were found with symptoms such as loss of strength or weakness (*p* = 0.002), headache (*p* = 0.00001), and fever (*p* < 0.00001), although these associations did not differ by gender. **Conclusions:** The outcomes of this study underscore the disparities in stroke types and risk factors between genders, providing valuable insights for the development of gender-specific approaches for stroke assessment and prevention among young individuals in Pakistan.

## 1. Introduction

Stroke remains one of the major causes of death and long-term disability worldwide, raising serious health concerns [1]. Since the condition typically affects the middle-aged and elderly, strokes in young adults are very rare [2]. The World Health Organization has defined stroke as a condition characterized by the rapid onset of clinical symptoms indicating a focal or global impairment of cerebral function. These symptoms typically last for more than 24 h or result in death, with no identifiable cause other than of vascular origin [3].

Strokes are grouped into two main groups: ischemic and hemorrhagic strokes. The Trial of Org 10172 in Acute Stroke Treatment (TOAST) classification system is the most widely used mechanistic sub-classification system for patients with ischemic stroke, defining five subtypes: (i) large-artery atherosclerosis, (ii) cardio embolic, (iii) small vessel occlusion, (iv) stroke of other determined etiology, and (v) stroke of undetermined etiology [4]. Hemorrhagic stroke may be further subdivided into intracerebral hemorrhage (ICH) and subarachnoid hemorrhage (SAH) [5].

Over the last decade, sex differences in stroke epidemiology, risk factors, management, and outcomes have been thoroughly investigated, and there is now enough evidence to suggest that stroke pathophysiology is gender-specific. The fundamental cause of sex-related gaps in stroke is variations in sex steroid hormones, notably the estrogen hormone. This theory is supported by sex differences in ischemic stroke in animal models [6]. Furthermore, the hormone estradiol in females dilates the vascular endothelium and increases blood flow, whereas testosterone in males constricts the endothelial and reduces blood flow [7]. Genetic and anatomical factors may influence sex variations in stroke incidence, etiology, and clinical outcomes [8].

The classic risk factors for stroke in the majority of people, such as high blood pressure, high cholesterol, obesity, diabetes mellitus, smoking, and heart disease, are also present in young adults, but there are other characteristics unique to the young population that can increase the risk of stroke: migraines, oral contraceptive use, pregnancy and the postpartum period, patent foramen ovale, and recreational drug use [9].

Young strokes occur frequently in patients aged 15 to 45, or in those under 50 [2]. Stroke prevalence ranges from 5 to 10% in developed nations and from 19 to 30% in developing nations for those under 45 years old [10]. Stroke is the second most prevalent cause of death worldwide, affecting 16.9 million people each year [11]. In comparison to the rest of the world, South Asia has a higher prevalence rate of stroke, younger ages at which stroke occurs, higher mortality rates, an increased number of modifiable risk factors for stroke, and several understudied non-conventional risk factors [12].

Pakistan, the sixth most populated country in the world, has insufficient population-based, rigorous data on the prevalence and risk factors of stroke [13]. A decade ago, in Karachi, a city in Pakistan, only two small-scale population-based studies were conducted. The prevalence of stroke reported in these studies was 4.8% [14] and 19.1% [15], which is the highest in the region.

An extensive, population-based investigation was required to determine the prevalence of stroke in Pakistan’s rural and urban areas. There is a lack of comprehensive data concerning sex disparities in stroke-related risk factors, clinical profiles, and both preventive and care strategies, especially regarding different types of strokes and their associated clinical and laboratory characteristics in young stroke patients. The objective of this study is to conduct a thorough analysis of gender differences and stroke subtypes with the symptoms, risk factors, and clinical and laboratory aspects of stroke, focusing specifically on young stroke patients. It is important to have comprehensive and detailed information on these variations to develop more effective strategies for preventing and managing stroke.

## 2. Methods

This retrospective, comparative study was carried out in a local hospital in Pakistan from August 2022 to March 2024. Patients who were admitted with a diagnosis of cerebrovascular accident based on clinical and radiological findings (non-contrast brain CT scan) were included in this study. All 185 patients were included based on the inclusion and exclusion criteria.

### 2.1. Inclusion Criteria

Young individuals with first-ever stroke who met the following criteria were eligible for inclusion: (1) age range of 18 to less than 45 years; (2) cerebral and hemorrhagic infarction confirmed by computed tomography or magnetic resonance imaging.

### 2.2. Exclusion Criteria

The following criteria were followed while excluding patients: transient ischemic attack; (1) venous infarction; (2) head trauma-related stroke; (3) iatrogenic stroke, including stroke resulting from major surgery, angiography, or carotid endarterectomy; (4) patients with recurrent stroke; (5) patients with severe stroke; or aphasia (6) patients who died as a result of their stroke.

### 2.3. Data Collection

Questionnaire responses were gathered from multiple sources, including a review of medical records, direct interviews during follow-up appointments, and telephone interviews for patients unable to attend in person. This study employed a patient questionnaire to collect subjective information directly from participants, while objective data, such as laboratory and imaging test results, were obtained separately from the patient’s medical records. We utilized the National Cholesterol Education Program (NCEP) Adult Treatment Panel III (ATP III) for the assessment of laboratory characteristics [16].

To maintain confidentiality, code numbers were used instead of patient names when entering the collected data into SPSS.

### 2.4. Statistical Analysis

The data gathered for this study were cleaned, processed, fed, and analyzed using SPSS software version 24.0. Categorical variables were compared using Pearson’s chi-squared test. The study participants were described using frequency, percentage, mean, and standard deviation. Variables with a *p*-value less than 0.05 are considered significant. The results are presented in the form of text and summary tables.

## 3. Results

### 3.1. Socio-Demographic Characteristics of the Study Population

**Figure 1:**
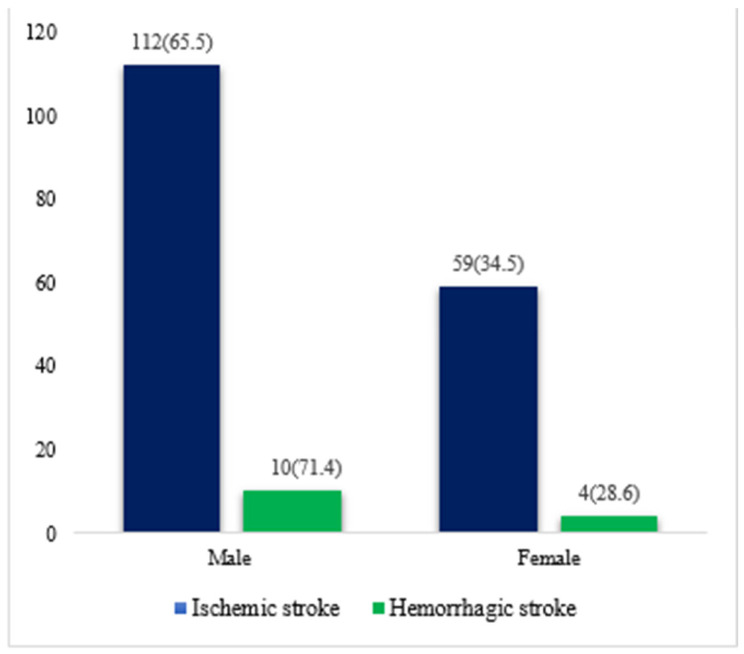
Distribution of stroke types by gender.

Out of the 185 study participants, 122 (65.9%) were male and 63 (34.1%) were female (Figure 1). The average age of the male patients was 13.36 ± 34.82 years, and the average age of the female patients was 15.26 ± 35.59 years. The age categories are presented in Table 1, revealing that within the 41–45 age category, male patients exhibit a higher incidence of stroke. The majority of patients, 171 (92.4%), experienced ischemic strokes, while 14 (7.6%) experienced hemorrhagic strokes. The stroke etiology, as per the TOAST criteria, is detailed in Table 1. Large-artery atherosclerosis was the most prevalent at 124 (67.0%), with cardiac embolism at 1 (0.5%) and small vessel disease at 25 (13.5%), making them less common. Hemorrhagic strokes were further categorized as intracerebral hemorrhage at seven (3.8%) and subarachnoid hemorrhage at seven (3.8%).

### 3.2. Characteristics and Symptoms by Gender in Young Stroke Patients

The distribution of clinical characteristics among genders is outlined in Table 2. The prevalence of hypertension, diabetes, obesity, and smoking was notably higher among males compared to females (*p* = 0.01 for smoking).

HDL was found to be more common among males than females, with statistically significant differences (*p* = 0.01 for HDL) (Table 3).

**Figure 2:**
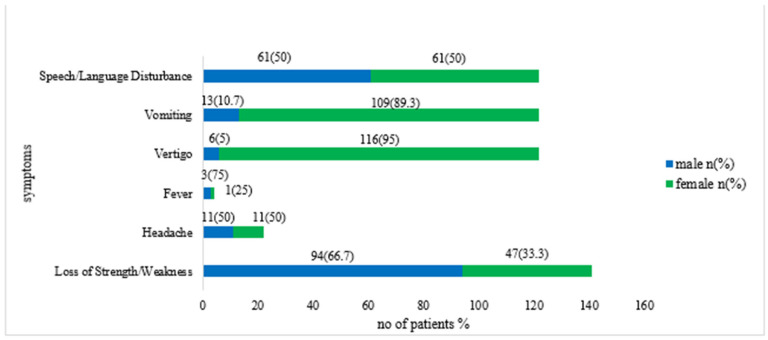
Distribution of symptoms by gender.

The variations in symptoms between males and females are presented in Figure 2. Upon admission to the hospital, the most frequently reported clinical presentation was loss of strength or weakness, observed in 94 males (66.7%) and 47 females (33.3%). Similarly, vomiting was predominantly reported by females, affecting 109 individuals (89.3%) versus 13 males (10.7%).

### 3.3. Comparative Analysis of Clinical, Laboratory Characteristics, and Symptoms by Stroke Type in Young Patients

The distribution of clinical characteristics among stroke types is outlined in Table 4. Hypertension (36.2), diabetes (26.5%), and obesity (30.8%), were more common in ischemic stroke than in hemorrhagic stroke, with no statistical significance.

The distribution of clinical characteristics among stroke types is outlined in Table 5. The table compares the distribution of various lipid profile components (LDL, HDL, cholesterol, and triglycerides) between patients with ischemic stroke and hemorrhagic stroke, which shows no statistical significance.

The differences in symptoms between ischemic and hemorrhagic stroke are presented in Table 6. The majority of patients diagnosed with ischemic stroke commonly present with symptoms such as loss of strength or weakness (73%), headaches (7.6%), and fever (2.1%), in comparison to those diagnosed with hemorrhagic stroke. The statistical analysis shows a significant difference in ischemic stroke with *p*-values of 0.002 for loss of strength or weakness, *p* = 0.00001 for headache, and *p* ≤ 0.00001 for fever.

## 4. Discussion

Stroke is a prevalent issue in developing countries; however, it is currently being given inadequate attention. Approximately 86% of all stroke-related fatalities globally occur in low- and middle-income countries [17]. Although there is no extensive epidemiological research available to ascertain Pakistan’s actual stroke incidence, the country’s estimated yearly incidence of stroke is 250/100,000 [18]. Based on our current understanding, this study is the first of its kind to explore gender differences in stroke subtypes, risk factors, clinical presentations, and laboratory results at a single stroke center in Pakistan.

The majority of patients, 171 (92.4%), experienced ischemic strokes, while 14 (7.6%) experienced hemorrhagic strokes. This finding aligns with multiple research findings that indicate that ischemic stroke is more common than hemorrhagic stroke. Research conducted in Libya, Africa, revealed that of their stroke cases, 23% were hemorrhagic stroke cases and 77% were ischemic stroke cases (including subarachnoid and intracerebral hemorrhagic stroke) [19]. In Morocco, there were 12.7% hemorrhagic strokes and 87.3% ischemic strokes, with a lower proportion of hemorrhagic stroke [20]. Another six-month study, conducted in the Neurology Department at the Mayo Hospital in Lahore from 6 February 2018 to 6 August 2018, showed that a significant number of patients with ischemic stroke are female [21]. The increased incidence of ischemic stroke may be ascribed to modifiable risk factors, including hypertension, diabetes, and lifestyle-related influences, which are prevalent within our population. Cultural and dietary practices, such as excessive salt consumption and insufficient physical activity, may further exacerbate vascular risk profiles.

In the present study, the prevalence of hypertension, diabetes, obesity, and smoking was notably higher in males compared to females. Smoking was identified as a significant risk factor among males. These findings are consistent with a study conducted in India, which demonstrated that the increased prevalence of risk factors such as obesity, diabetes, hypertension, alcoholism, and sedentary lifestyles is strongly associated with the rising incidence of stroke cases [22]. The higher prevalence of smoking among males, along with cultural acceptance of this behavior, notably heightens their vascular risk. Conversely, research indicates that women may have a greater prevalence of hypertension and atrial fibrillation. This could be associated with hormonal changes, especially following menopause, as well as disparities in healthcare access. Furthermore, other studies have indicated that while hypertension and atrial fibrillation are more prevalent among women, smoking and vascular diseases are more commonly observed in men. These results corroborate previous research that emphasizes sex-based differences in stroke risk factors [23].

Numerous studies have indicated that reduced levels of total cholesterol (TC) and higher-density lipoprotein cholesterol (HDL) are correlated with an elevated risk of cerebral hemorrhage [24]. In our analysis, the HDL levels were *p* = 0.018. This observed gender-based discrepancy implies potential variations in lipid metabolism and cardiovascular risk profiles, which may influence the differing presentations and outcomes of strokes between males and females.

Vertigo is characterized as a particular kind of vertigo that combines nausea/vomiting, unsteadiness in walking, and an uncomfortable distortion of static gravitational direction. It makes up 3–5% of all visits and is the third most frequent main symptom to appear in general medicine clinics [25]. In the present study, a significantly higher number of females presented with vertigo (116 or 62.7%). This finding highlights the potential existence of gender-related differences in the prevalence or reporting of vertigo, necessitating further investigation into the biological, hormonal, or psychosocial factors that may contribute to this disparity.

This study further found the prevalence of hypertension (36.2%), diabetes (26.5%), and obesity (30.8%) in ischemic stroke compared to hemorrhagic stroke to reflect existing research trends, although our findings did not reach statistical significance. This correlation is supported by studies such as those conducted by Willmot et al. (2004) [26] and Strazzullo et al. (2010) [27], which emphasize the role of obesity in increasing stroke risk through mechanisms such as insulin resistance and systemic inflammation. Despite the lack of statistical significance in our study, these connections underscore the importance of effectively managing these risk factors to prevent ischemic strokes.

The lipid profiles in stroke etiology are compared between ischemic and hemorrhagic strokes. It is commonly believed that low-density lipoprotein (LDL) cholesterol is linked to atherosclerosis and the risk of ischemic stroke. However, our study did not find a significant difference in LDL levels between patients with ischemic and hemorrhagic strokes. This aligns with the INTERSTROKE study by O’Donnell et al. (2010) [28], which emphasized the complex and sometimes unclear role of LDL in different types of strokes. Research, such as the Framingham Heart Study conducted by Castelli et al. (1986) [29], has shown a connection between high-density lipoprotein (HDL) levels and the risk of ischemic stroke. Despite these findings, our study did not identify a significant disparity in HDL levels between patients with ischemic and hemorrhagic strokes. Our study found that there was no significant variation in total cholesterol levels between patients who experienced ischemic strokes and those who had hemorrhagic strokes. This is in line with previous research, such as the Copenhagen City Heart Study (Nordestgaard et al., 2007) [30], which also found no significant disparities in cholesterol levels between different types of strokes. This suggests that other factors may be more influential in the development of strokes.

Triglycerides, a type of fat present in the bloodstream, have been linked to cardiovascular events, including strokes. Higher levels of triglycerides have been associated with an increased risk of ischemic strokes, as indicated by the Northern Manhattan Stroke Study [31]. This study did not identify any statistically significant variance in triglyceride levels between patients with ischemic and hemorrhagic strokes. This outcome is consistent with prior research, such as the study conducted by Amarenco et al. in 2006 [32], which indicated that while triglycerides play a role in cardiovascular risk factors, their specific impact on distinguishing between types of strokes remains uncertain. Analysis comparing symptoms between ischemic and hemorrhagic strokes, as presented in Table 6, reveals noteworthy distinctions in their clinical manifestations.

The high occurrence of weakness or loss of strength in ischemic stroke patients aligns with studies suggesting that motor impairments are common following ischemic strokes due to localized brain ischemia [33]. The significant difference in headache occurrence (*p* = 0.00001) supports the assertion that headaches are more prevalent and severe in hemorrhagic strokes, likely attributable to elevated intracranial pressure [34]. Furthermore, the increased incidence of fever in patients with ischemic stroke (*p* < 0.00001) may suggest the presence of an inflammatory reaction linked to cerebral infarction [35]. The variation in symptom prevalence between ischemic and hemorrhagic strokes highlights the distinct pathophysiological pathways involved in each stroke subtype.

Consequently, individualized treatment strategies specific to the type of stroke are warranted. Recognizing unique symptom patterns can aid healthcare professionals in timely and accurate stroke diagnosis, potentially resulting in improved patient outcomes through tailored interventions.

## 5. Limitations

This study presents several limitations that should be considered when interpreting its findings. Firstly, it is a single-center investigation, which may restrict the generalizability of the results to wider populations. The outcomes are derived from a specific cohort of patients treated at one institution, potentially introducing selection bias and possibly not reflecting the full diversity of stroke patients in other geographic regions or healthcare settings.

Furthermore, the retrospective design of this study presents inherent challenges in establishing causality. The reliance on historical data and medical records may contribute to incomplete or inconsistent information, particularly concerning risk factors that were not systematically evaluated or documented. Examples of these risk factors include genetic predispositions, persistent foramen ovale (PFO), and antiphospholipid syndrome. The absence of comprehensive information regarding these common risk factors for young stroke further restricts the overall scope of the findings.

Additionally, the limited sample size of this study may hinder the statistical power necessary to identify more nuanced associations or differences among subgroups. Although the research offers valuable insights into ischemic stroke patterns, the relatively small number of participants, particularly among younger individuals, diminishes the robustness of the conclusions regarding specific risk factors.

To address these gaps, future studies should employ comprehensive diagnostic methods, including thrombophilia screening, genetic testing, and advanced cardiac imaging techniques. These investigations will clarify the role of specific risk factors in the development of strokes, particularly in younger adults, and contribute to a more thorough understanding of stroke etiology across all age groups.

## 6. Conclusions

In conclusion, the findings of this study contribute to a deeper comprehension of the variety of risk factors, stroke types, diagnostic methods, and gender disparities in stroke among young adults seen at a hospital in Pakistan. It is imperative to conduct large community-based prospective cohort studies with sufficient statistical power to corroborate these results. This study’s identification of gender disparities in risk factors and causes of stroke offers valuable insights for developing gender-specific strategies for stroke assessment and prevention among young adults in Pakistan. Our findings highlight the need for validation through a prospective study using standardized methods. Furthermore, it is essential to undertake extensive longitudinal studies on a larger scale to gain a comprehensive understanding of the underlying causes and risk factors associated with stroke occurrence in younger patients.

## Figures and Tables

**Table 1:** Baseline demographic and clinical information on stroke patients overall and by sex.

Demographics	All n (%)	Gender		*p*-Value
	All	Male n (%)	Female n (%)	
	185	122 (65.9)	63 (34.1)	
Age in years: mean + (SD)	M: 39.1 SD: 5.5	M: 39.3 SD: 5.3	M: 38.7 SD: 5.9	
Stay in hospital (in days)	M:15.9 SD:37.6	M: 13.3 SD 34.8	M: 15.2 SD 35.5	
Age	185	122 (65.9)	63 (34.1)	
18–25		2 (1.0)	4 (2.1)	
26–30		5 (2.7)	1 (0.5)	
31–35		19 (1.02)	5 (2.7)	0.21
36–40		36 (19.4)	21 (11.3)	
41–45		60 (32.4)	32 (17.2)	
Ischemic stroke (TOAST)	171 (92.4)	112 (65.5)	59 (34.5)	
Large-artery atherosclerosis (LAA)	124 (67.0)	82 (44.3)	42 (22.7)	
Cardioembolism (CA)	1 (0.5)	0	1 (0.5)	0.39
Small-vessel occlusion (SVD)	25 (13.5)	16(8.6)	9 (4.8)	
Stroke of determined cause	9 (4.9)	4(2.1)	5 (2.7)	
Stroke of undetermined cause	12 (6.5)	10(5.4)	2 (1.0)	
Hemorrhagic stroke	14 (7.6)	10(71.4)	4 (28.6)	
Intracerebral hemorrhage	7 (3.8)	6 (3.2)	1 (0.5)	0.23
Subarachnoid hemorrhage	7 (3.8)	4 (2.1)	3 (1.6)	

**Table 2:** Clinical characteristics of young stroke patients by gender.

	Male n (%)	Female n (%)	*p*-Value
**Hypertensive**			
Yes	47 (25.4)	27 (14.6)	0.5
No	75 (40.5)	36 (19.5)	
**Diabetes**			
Normal	57 (30.8)	24 (13)	
Pre-diabetic	14 (7.6)	12 (6.5)	0.23
Diabetic	32 (17.3)	21 (11.4)	
Unknown	19 (10.2)	6 (3.2)	
**Body mass index (kg/m^2^)**			
Underweight: BMI < 18.5 kg/m^2^	39 (21.1)	20 (10.8)	
Normal weight: BMI 18.5–24.99 kg/m^2^	40 (21.6)	23 (12.4)	0.60
Overweight: BMI 25–29.99 kg/m^2^	36 (19.5)	19 (10.3)	
Obese: BMI ≥ 30 kg/m^2^	7 (3.8)	1 (0.5)	
**Smoking**			
Yes	15 (8.1)	107 (57.8)	0.014 *
No	1 (0.5)	62 (33.5)	

* means highly significant values.

**Table 3:** Laboratory characteristics of young stroke patients by gender.

Laboratory Characteristics	Male n (%)	Female n (%)	*p*-Value
**LDL (** **mg/dL)**			
Optimal (<100 mg/dL)	32 (17.3)	21 (11.3)	
High (>160 mg/dL)	12 (6.5)	5 (2.7)	
Borderline (130–160 mg/dL)	25 (13.5)	5 (2.7)	0.22
Above optimal (<130 mg/dL)	26 (14.1)	14 (7.6)	
Unknown	27 (14.6)	18 (9.7)	
**HDL (mg/dL)**			
Major risk factor for heart diseases (<40 mg/dL)	70 (37.8)	23 (12.4)	
Negative risk for heart disease (≥ 60 mg/dL)	47 (25.4)	38 (20.5)	0.018 *
Unknown	5 (2.7)	2 (1.1)	
**Cholesterol (mg/dL)**			
Desirable (<200 mg/dL)	69 (37.2)	38 (20.5)	
Borderline (200–239 mg/dL)	17 (9.1)	6 (3.2)	0.50
High (>240 mg/dL)	8 (4.3)	2 (1.1)	
Unknown	25 (13.5)	17 (9.1)	
**Triglycerides (mg/dL)**			
Normal (<150 mg/dL)	57 (30.8)	29 (15.7)	
Borderline (150–190 mg/dL)	9 (4.9)	6 (3.2)	0.60
High (200–499 mg/dL)	24 (13)	8 (4.3)	
Unknown	32 (17.3)	20 (10.8)	

* means highly significant values.

**Table 4:** Clinical characteristics of young patients by stroke type.

Clinical Characteristics	Ischemic Stroke	Hemorrhagic Stroke	*p*-Value
**Hypertensive**			0.427
Yes	67 (36.2)	7 (3.8)	
No	104 (56.2)	7 (3.8)	
**Diabetes**			
Normal	74 (40)	7 (3.8)	0.397
Pre-diabetic	26 (14.0)	0	
Diabetic	49 (26.5)	4 (2.1)	
Unknown	22 (11.9)	3 (1.6)	
**Body mass index (kg/m^2^)**			
Underweight: BMI < 18.5 kg/m^2^	8 (4.3)	0	0.70
Normal weight: BMI 18.5–24.99 kg/m^2^	56 (30.2)	3 (1.6)	
Overweight: BMI 25–29.99 kg/m^2^	50 (27.0)	5 (2.7)	
Obese: BMI ≥ 30 kg/m^2^	57 (30.8)	6 (3.2)	
**Smoking**			
Yes	16 (8.6)	0	0.77
No	155 (83.8)	14 (17.6)	

**Table 5:** Laboratory characteristics of young patients by stroke type.

Laboratory Characteristics	Ischemic Stroke	Hemorrhagic Stroke	*p*-Value
**LDL** **(mg/dL)**			
Optimal (<100 mg/dL)	51 (27.6)	2 (1.1)	0.15
High (>160 mg/dL)	17 (9.2)	0	
Borderline (130–160 mg/dL)	28 (15.3)	2 (1.1)	
Above optimal (<130 mg/dL)	37 (20)	3 (1.6)	
Unknown	38 (20.5)	7(3.8)	
**HDL (mg/dL)**			
Major risk factor for heart diseases (<40 mg/dL)	89 (48.1)	4 (2.2)	0.127
Negative risk for heart disease	75 (40.5)	10	
Unknown	7 (3.8)	0	
**Cholesterol (mg/dL)**			
Desirable (<200 mg/dL)	102 (55.1)	5 (2.7)	0.87
Borderline (200–239 mg/dL)	22 (11.9)	1 (0.5)	
High (>240 mg/dL)	9 (4.9)	1 (0.5)	
Unknown	35 (19)	7 (3.8)	
**Triglycerides (mg/dL)**			
Normal (<150 mg/dL)	80 (43.2)	6 (3.2)	0.191
Borderline (150–190 mg/dL)	15 (8.1)	0	
High (200–499 mg/dL)	31 (16.7)	1 (0.5)	
Unknown	45 (24.3	7 (3.8)	

**Table 6:** Clinical presentations of stroke at the time of admission to the neurology ward.

Symptoms	Ischemic Stroke n (%)	Hemorrhagic Stroke n (%)	*p*-Value
**Loss of Strength/Weakness**			
Yes	135 (73)	6 (3.2)	0.002 *
No	36 (19.4)	8 (4.3)	
**Headache**			
Yes	14 (7.6)	8 (4.3)	0.00001 *
No	157 (84.9)	6 (3.2)	
**Fever**			
Yes	4 (2.1)	0	<0.00001*
No	167 (90.2)	14 (7.6)	
**Vertigo**			
Yes	89 (48.1)	5 (2.7)	0.08
No	82 (44.3)	9 (4.9)	
**Vomiting**			
Yes	18 (9.7)	3 (1.6)	0.21
No	153 (82.7)	11 (5.9)	
**Speech/Language Disturbance**			
Yes	89 (48.1)	5 (2.7)	0.24
No	82 (44.3)	9 (4.9)	

* means highly significant values.

## Data Availability

The data presented in this study are available on request from the corresponding author. The data collected from patients are confidential, so it cannot be made available online, but it can be provided on demand under prescribed rules and regulations.
